# Implementation of Early Next-Generation Sequencing for Inborn Errors of Immunity: A Prospective Observational Cohort Study of Diagnostic Yield and Clinical Implications in Dutch Genome Diagnostic Centers

**DOI:** 10.3389/fimmu.2021.780134

**Published:** 2021-12-21

**Authors:** Kim Elsink, Manon M. H. Huibers, Iris H. I. M. Hollink, Annet Simons, Evelien Zonneveld-Huijssoon, Lars T. van der Veken, Helen L. Leavis, Stefanie S. V. Henriet, Marcel van Deuren, Frank L. van de Veerdonk, Judith Potjewijd, Dagmar Berghuis, Virgil A. S. H. Dalm, Clementien L. Vermont, Annick A. J. M. van de Ven, Annechien J. A. Lambeck, Kristin M. Abbott, P. Martin van Hagen, Godelieve J. de Bree, Taco W. Kuijpers, Geert W. J. Frederix, Mariëlle E. van Gijn, Joris M. van Montfrans, the Genetics First for Primary Immunodeficiency Disorders Consortium, Aerde van, KJ

**Affiliations:** ^1^ Department of Pediatric Immunology and Infectious Diseases, Wilhelmina’s Children Hospital, University Medical Center Utrecht, Utrecht University, Utrecht, Netherlands; ^2^ Department of Genetics, Division Laboratories, Pharmacy and Biomedical Genetics, University Medical Center Utrecht, Utrecht University, Utrecht, Netherlands; ^3^ Department of Clinical Genetics, Erasmus Medical Center, Erasmus University Rotterdam, Rotterdam, Netherlands; ^4^ Department of Human Genetics, Nijmegen Center for Molecular Life Sciences, Radboud University Medical Centre, Radboud University, Nijmegen, Netherlands; ^5^ Radboud Institute for Oncology, Radboud University Medical Center, Radboud University, Nijmegen, Netherlands; ^6^ Department of Genetics, University Medical Center Groningen, University of Groningen, Groningen, Netherlands; ^7^ Department of Rheumatology and Clinical Immunology, University Medical Center Utrecht, Utrecht University, Utrecht, Netherlands; ^8^ Department of Pediatric Infectious Diseases and Immunology, Amalia’s Children Hospital, Radboud University Nijmegen Medical Centre, Radboud University, Nijmegen, Netherlands; ^9^ Department of Internal Medicine, Radboud University Medical Center, Radboud Center for Infectious Diseases, Nijmegen, Netherlands; ^10^ Department of Nephrology and Clinical Immunology, Maastricht University Medical Center, Maastricht University, Maastricht, Netherlands; ^11^ Willem-Alexander Children’s Hospital, Department of Pediatrics, Leiden University Medical Center, Leiden University, Leiden, Netherlands; ^12^ Department of Internal Medicine, Division of Allergy & Clinical Immunology; Department of Immunology, Erasmus University Medical Center Rotterdam, Erasmus University Rotterdam, Rotterdam, Netherlands; ^13^ Department of Pediatric Infectious Diseases, Immunology and Rheumatology, Sophia Children’s Hospital, Erasmus Medical Center, Erasmus University Rotterdam, Rotterdam, Netherlands; ^14^ Department of Internal Medicine and Allergology, Rheumatology and Clinical Immunology, University Medical Center Groningen, Groningen, Netherlands; ^15^ Department of Laboratory Medicine, University Medical Center Groningen, University of Groningen, Groningen, Netherlands; ^16^ Department of Internal Medicine, Institute for Infection and Immunity, Amsterdam University Medical Center, University of Amsterdam, Amsterdam, Netherlands; ^17^ Department of Pediatric Hematology, Immunology and Infectious Diseases, Emma Children’s Hospital, Amsterdam University Medical Center, University of Amsterdam, Amsterdam, Netherlands; ^18^ Julius Center for Health Sciences and Primary Care, University Medical Centre Utrecht, Utrecht, Netherlands

**Keywords:** next-generation sequencing, inborn errors of immunity, diagnostic yield, gene panel, clinical implication

## Abstract

**Objective:**

Inborn errors of immunity (IEI) are a heterogeneous group of disorders, affecting different components of the immune system. Over 450 IEI related genes have been identified, with new genes continually being recognized. This makes the early application of next-generation sequencing (NGS) as a diagnostic method in the evaluation of IEI a promising development. We aimed to provide an overview of the diagnostic yield and time to diagnosis in a cohort of patients suspected of IEI and evaluated by an NGS based IEI panel early in the diagnostic trajectory in a multicenter setting in the Netherlands.

**Study Design:**

We performed a prospective observational cohort study. We collected data of 165 patients with a clinical suspicion of IEI without prior NGS based panel evaluation that were referred for early NGS using a uniform IEI gene panel. The diagnostic yield was assessed in terms of definitive genetic diagnoses, inconclusive diagnoses and patients without abnormalities in the IEI gene panel. We also assessed time to diagnosis and clinical implications.

**Results:**

For children, the median time from first consultation to diagnosis was 119 days versus 124 days for adult patients (U=2323; p=0.644). The median turn-around time (TAT) of genetic testing was 56 days in pediatric patients and 60 days in adult patients (U=1892; p=0.191). A definitive molecular diagnosis was made in 25/65 (24.6%) of pediatric patients and 9/100 (9%) of adults. Most diagnosed disorders were identified in the categories of immune dysregulation (n=10/25; 40%), antibody deficiencies (n=5/25; 20%), and phagocyte diseases (n=5/25; 20%). Inconclusive outcomes were found in 76/165 (46.1%) patients. Within the patient group with a genetic diagnosis, a change in disease management occurred in 76% of patients.

**Conclusion:**

In this cohort, the highest yields of NGS based evaluation for IEI early in the diagnostic trajectory were found in pediatric patients, and in the disease categories immune dysregulation and phagocyte diseases. In cases where a definitive diagnosis was made, this led to important disease management implications in a large majority of patients. More research is needed to establish a uniform diagnostic pathway for cases with inconclusive diagnoses, including variants of unknown significance.

## Introduction

Inborn errors of immunity (IEI) are a heterogeneous group of inherited disorders affecting different components of the immune system (1, 2). Clinical manifestations include increased susceptibility to infections, failure to thrive, autoimmunity, auto-inflammatory diseases, and/or malignancies. Severity of these manifestations and of disease related complications range from mild, with relatively little disease burden, to severe life-threatening complications. Primary antibody deficiencies usually give rise to an increased risk of sino-pulmonary tract infections and are often treated with prophylactic antibiotics and/or immunoglobulin replacement therapy (IGRT). Other IEI include, for example, various types of (severe) combined immunodeficiency disorder (SCID or CID) and hemophagocytic lymphohistiocytosis (HLH), which require allogeneic hematopoietic stem cell transplantation (HSCT) or autologous hematopoietic stem cell based gene therapy.

Next-generation sequencing (NGS) has proven to be useful in the diagnostic evaluation of different diseases (3–6). In the field of immunology, as of 2021, over 450 IEI related genes have been reported, and targeted next-generation sequencing became integrated in the routine diagnostic process (7–9). Different studies have been published on the diagnostic yield of NGS in subtypes of IEI and identification of novel disease-causing genes, reporting diagnostic yields from 15 to 79% (10–16). Early diagnosis in IEI enables timely referral for diagnosis treatment and follow up, resulting in improved treatment outcome in several types of immune deficiencies. For example, in the setting of SCID, early detection enables timely referral for HSCT, which was shown to improve treatment outcome and to reduce health care costs (17).

Although NGS is widely used in the evaluation of IEI, its overall diagnostic yield and optimal timing in the diagnostic work up still have to be determined. Moreover, prospective data on the extent of changes in clinical management and prognosis are still lacking. In 2017, consensus was reached among all Dutch genome diagnostic laboratories to adopt a uniform IEI gene panel, based on updates from the International Union of Immunological Societies (IUIS), for nationwide use in the evaluation of IEI (18, 19). The implementation of this uniform IEI panel as a routine diagnostic tool provided the opportunity to prospectively study the diagnostic yield and clinical implications in a daily practice setting in a prospective cohort of 165 patients referred for early NGS.

## Methods and Materials

### Study Type

We performed a prospective observational cohort study in patients referred for evaluation of an inborn error of immunity (IEI).

### Patient Selection and Data Collection

We selected data from 165 patients who had a suspicion of an IEI and were referred by a clinical immunologist for next-generation sequencing (NGS) within one of seven Dutch academic medical centers from 2017 to 2020, irrespective of age and sex. Patients were selected when a clinical suspicion of IEI existed, based on clinical manifestations, severity of infections, severity of immune dysregulation and/or laboratory abnormalities. After inclusion, patients were subsequently categorized according to International Union of Immunological Society (IUIS) classification (20). We excluded patients who had undergone prior NGS based panel evaluation for IEI.

We extracted data on clinical presentation, laboratory evaluation, genetic testing and management implications from the electronic patient files. We separately collected the date of suspicion of IEI, defined as date of first outpatient visit or first clinical consultation for IEI, and date of diagnosis, defined as the date of the genetic test result provided by the clinical laboratory geneticist.

The study was approved by the Medical Ethical Board of the Erasmus MC. Informed consent to perform the descriptive analyses in this cohort study was waived by the Medical Ethical Board of the Erasmus MC.

### Next-Generation Sequencing

Patients in the study underwent either whole-exome sequencing or whole-genome sequencing, depending on the specific medical center where the genetic testing was performed, followed by targeted and nationwide uniform IEI gene panel analysis (19).

### NGS Pipeline

Genetic evaluation of the samples was performed according to the diagnostic pipeline of the genome centers where the analyses were performed, as described by Elsink et al. (2021) (19).

In short, 65/165 samples were sequenced using a WGS approach, with a mean coverage depth of 30x (19). For the bioinformatics analyses of these samples, in-house developed pipelines were used, with bwa-mem for mapping and GATK (v3.8-1-0-gf15c1c3ef and v 3.7) for variant calling. For variant annotation, Alissa Interpret (Agilent Technologies) and Alamut Visual (Sophia Genetics) were used.

A total of 100/165 samples were sequenced using a WES approach, with a mean coverage depth of >50 to >75x, depending on the genome diagnostic center where sequencing was performed (19). For the bioinformatics, aGATK HaplotypeCaller (v.3.4-46) for variant calling and in-house pipeline using bwa-mem 0.7.13-r1126 for mapping and Picard Mark duplicates and GATK HaplotypeCaller (v3.7-0-gcfedb67) for variant calling was used. For variant annotation, Alissa Interpret (Agilent Technologies) and Alamut Visual were used, or an in-house pipeline for exome analysis (19).

### Gene Panel

A uniform IEI gene panel was designed by the three participating diagnostic centers. During the study period, and following international reporting on IEI related genes, the panel was updated by three-monthly consensus meetings, primarily based on annual updates of the IUIS classification (20) and on conference proceedings. Therefore, the applied gene panel depended on the specific timing that a patient underwent NGS. The gene panel consisted of 360 genes at the start of the study period (2017) and 424 genes by the end of the study (2020) (**Table S1**).

### Classification and Reporting of Genetic Findings

Variant interpretation was performed according to the ACMG guidelines (21) and variants were classified as pathogenic, likely pathogenic, unknown significance, likely benign and benign.

Variants classified pathogenic (class 5) and likely pathogenic (class 4) were reported to the referring clinician. The variants of unknown significance (class 3) were reported based on clinical relevance. The relevance of the variants of unknown significance was discussed in center based multidisciplinary meetings with clinical laboratory geneticists, clinical geneticists, immunologists, physicians and other experts as required based on the clinical situation. For analysis purposes, we divided the genetic findings into one of the following three categories: conclusive genetic diagnosis, inconclusive outcome (including risk factors, carrierships and VUS), or no abnormalities in IEI panel.

#### Conclusive Genetic Diagnosis

A genetic diagnosis was defined as the presence of one heterozygous (likely) pathogenic variant in diseases with autosomal dominant inheritance or when a (likely) pathogenic hemizygous variant was identified in case of X-linked (recessive) inheritance. In case of diseases with a recessive inheritance, a genetic diagnosis was considered confirmed if homozygosity or compound heterozygosity for the (likely) pathogenic variants were found.

#### Inconclusive Outcomes

##### Risk Factors

Risk factors were defined as variants that might contribute to the disorder in a multifactorial context. These variants are not the only cause of the disorder and do not display a monogenic inheritance pattern. This is the case when the frequency of variants in the unaffected general population exceeds the expected frequency for a rare monogenic disorder. They are also referred to as variants with reduced penetrance (22).

##### Carrierships

Carrierships were defined as the presence of one (likely) pathogenic variants in diseases with autosomal recessive inheritance that are unlikely to contribute to the phenotype of patient. This also includes cases in which a second variant was missed due to technical issues.

##### Variants of Unknown Significance (VUS)

A VUS was defined as variant with unknown clinical significance. For analysis purposes, we clustered all VUS separately from other inconclusive outcomes.

#### No Abnormalities Identified in IEI Panel

When no (likely) pathogenic variants or variants of unknown significance were identified by the clinical laboratory geneticists, the outcome was categorized as ‘no abnormalities in IEI gene panel’. Incidental findings were reported when identified.

After bioinformatic analysis, filtering and subsequent classification of the variants, the potential causative gene variants were reported to the referring clinician.

### Outcome Measures

#### Time to Diagnosis

The time to diagnosis was defined as the time between the first consultation for the suspicion of IEI and the genetic diagnosis. We also measured turn-around time (TAT) for NGS based IEI panel diagnostics, which was defined as the time between the arrival of the blood sample in the laboratory and the result of the genetic evaluation.

#### Genetic Diagnosis per Disease Category

First, we assessed the number of patients that were provided with a conclusive genetic diagnosis, an inconclusive outcome and a genotype without abnormalities in the gene panel. Next, we categorized these patients in IEI subcategories using the IUIS classification. In the conclusive genetic diagnosis group, we reported on the clinical implications for each specific patient.

### Statistical Analysis

For continuous variables that were normally distributed, mean and range were reported. Median and interquartile range were used in data with a skewed distribution. A Mann-Whitney U test was performed to compare differences in continuous variables between the groups with and without a conclusive genetic diagnosis, and between WES and WGS. A chi-squared test was applied to compare diagnostic yield between patients referred for WES or WGS. Analyses were performed by SPSS software version 26.0 (IBM, NY, USA).

## Results

### Demographics

Overall, 165 patients were enrolled in the study between 2017 and 2020 (**Table S2**). The cohort consisted of 74 males (44.8%) and 91 females (55.2%). The age range of the study population was 0 to 87 years. 65/165 patients (39.4%) were under the age of 18. The median age at the moment of the first outpatient visit or clinical consultation for a suspicion of IEI was 21 years (IQR 38 years).

Patients were clustered according to their clinical presentations in one of 11 IEI categories based on the IUIS classification (20). Most patients were referred for NGS based on clinical manifestations compatible with antibody deficiencies (n=47; 28.5%), immune dysregulation (n=35; 21.2%), combined immunodeficiencies with associated features (n=25; 15.2%) or autoinflammatory diseases (n=14;8.5%).

### NGS Technique and Time to Diagnosis

In 65/165 patients, the sequencing data used for the IEI panel analysis were generated using a WGS based approach, versus 100/165 patients using a WES based technique. Of the patients with a conclusive genetic diagnosis, 11 out of 65 (16.9%) were sequenced by WGS, and 14 out of 100 (14%) were analyzed by WES based approach (X^2^(1) = 0.262; p = 0.609).

Overall, the median time to diagnosis was 121 days (IQR 282 days, range 6 to 6801 days), with a lower mean time to diagnosis in patients under the age of 18 (119 vs 124 days). The range of time to diagnosis was 6 to 2314 days in children and 33 to 6801 days in adults. The overall median TAT was 59 days (IQR 42 days). Median TAT was lower in children (56 days) than in adults (60 days), ranging from 6 to 94 days and 23 to 96 days, respectively. A Mann-Whitney U test did not show a statistically significant difference between children and adults in time to diagnosis (U=2323; p=0.644) and turn-around time (U=1892; p=0.191). The median TAT was lower in WES (55 days, IQR 30) than in WGS (49 days, IQR 30). Differences in turn-around time between WES and WGS were not statistically significant (U=2980;p=0.807).

### NGS Outcome and Diagnostic Yield

#### Conclusive Genetic Diagnosis

**Figure 1:**
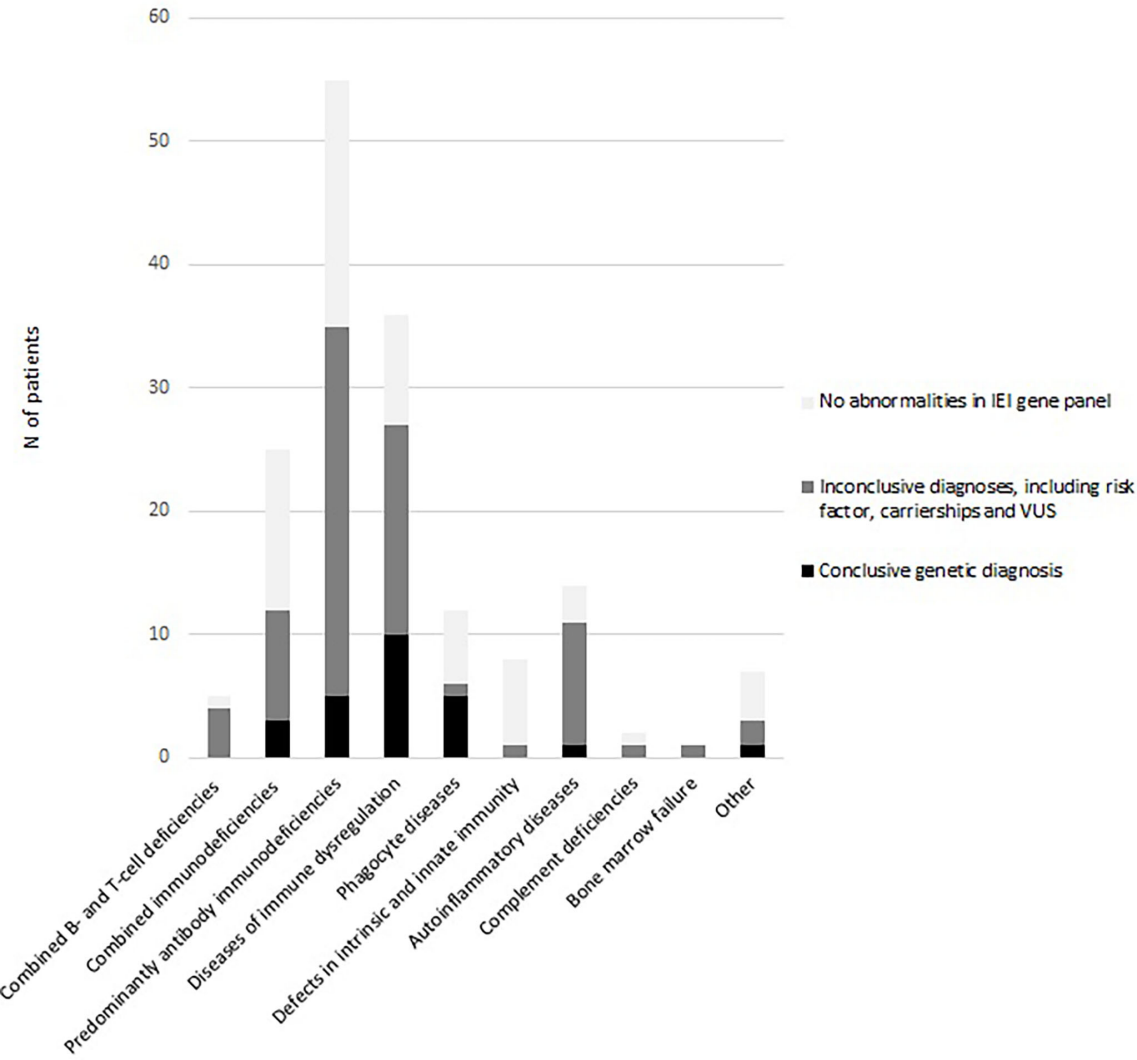
NGS outcomes per IUIS diagnosis category.

As shown in **Table 1**, a conclusive genetic diagnosis of IEI was made in 25/165 patients (15.2%). The overall yield in the age group <18 years was 24.6% (16/65), versus 9% in the adult patients (9/100). At the same time, the median age within the group with conclusive genetic diagnosis was 5 years (IQR 19 years) versus 27 years (IQR 37 years) in the group without conclusive diagnosis (U=874; p<0.05). As shown in **Figure 1**, the diagnostic yield was highest in in the disease categories of phagocyte diseases (n=5/12; 41.7%) and immune dysregulation (n=10/36; 27.8%)

**Table 1:** Outcomes of NGS IEI panel diagnostics, according to IUIS classification (23).

	Number of conclusive genetic diagnosis, n (% of total patients)	Inconclusive outcomes			No abnormalities in IEI gene panel	Overall diagnostic yield per IUIS disease category (%)	Total, n (%)
		Risk factor, n (%)	Carriership, n (%)	VUS, n (%)			
Combined B- and T-cell deficiencies	0 (0)	2 (1.2)	0 (0)	2 (1.2)	1 (0.6)	0	5 (3.0)
Combined immunodeficiencies with syndromic or associated features	3 (1.8)	1 (0.6)	5 (3.0)	3 (1.8)	13 (7.9)	3/25 (12)	25 (15.2)
Predominantly antibody deficiencies	5 (3.0)	8 (4.9)	10 (6.1)	12 (7.3)	20 (12.1)	5/55 (9.1)	55 (33.3)
Diseases of immune dysregulation	10 (6.1)	1 (0.6)	5 (3.0)	11 (6.7)	9 (5.5)	10/26 (27.8)	36 (21.8)
Phagocyte diseases	5 (3.0)	0 (0)	0 (0)	1 (0.6)	12 (7.3)	5/12 (41.7)	12 (7.3)
Defects in intrinsic and innate immunity	0 (0)	0 (0)	1 (0.6)	0 (0)	8 (4.8)	0	8 (4.8)
Autoinflammatory diseases	1 (0.6)	1 (0.6)	2 (1.2)	7 (4.2)	14 (8.5)	1/14 (7.1)	14 (8.5)
Complement deficiencies	0 (0)	0 (0)	1 (0.6)	0 (0)	1 (0.6)	0	2 (1.2)
Bone marrow failure	0 (0)	0 (0)	0 (0)	1 (0.6)	0 (0)	0	1 (0.6)
Other	1 (0.6)	0 (0)	1 (0.6)	1 (0.6)	4 (2.4)	1/7 (14.3)	7 (4.2)
Total N (%)	25 (15.2)	13 (7.9)	25 (15.2)	38 (23)	64 (38.8)	25	165 (100)

Specifically, most patients clustered within the immune dysregulation category were diagnosed with familial hemophagocytic lymphohistiocytosis (FHL) type 2 (n=3/10;30%), type 3 (n=2/10; 20%) and type 4 (n=1/10; 10%), based on homozygotic pathogenic variants in *PRF1*, *UNC13D*, and *STX11*, respectively (**Table S3**). Other diseases identified within this category included two cases of autoimmune lymphoproliferative syndrome (ALPS), both based on pathogenic *FAS* variants.

Patients with a conclusive diagnosis from the antibody deficiency category were diagnosed with activated PI3K-delta syndrome (APDS1) (n=2/5; 40%), X-linked agammaglobulinemia (XLA) (n=2/5; 40%) and AID deficiency (n=1/5; 20%), based on pathogenic variants in *PIK3CD*, *BTK* and *AICDA*, respectively.

The diagnoses clustered within the phagocyte diseases consisted of autosomal recessive chronic granulomatous disease (AR-CGD) (n=2/5; 40%), G6PD deficiency (n=2/5;40%) and MKL1 deficiency (n=1/5; 20%), based on pathogenic variants in *NCF1*, *G6PD* and *MKL1*, respectively.

#### Clinical Implications or Follow Up

Overall, the result of the NGS based IEI panel led to a direct change in disease management in 19/25 (76%) of patients in whom a conclusive genetic diagnosis was made. In cases without a direct change in disease management, the NGS outcome led to confirmation, continuation and/or optimization of existing therapy. As shown in **Table S3**, the outcome of the NGS marked the end of diagnostic trajectory in 21/25 (84%) patients. In two cases, the NGS evaluation led to further evaluation by a trio WES evaluation, which led to the genetic diagnosis of Gaucher disease in one case. In the other case, this led to the identification of pathogenic *G6PD* variants, that did not fully explain the clinical phenotype of the specific patient (**Table S3**).

In 9/25 (36%) of cases with a conclusive diagnosis, the genetic diagnosis led to an HSCT indication. This was the case for 6 patients with FHL, and for patients with MKL1 deficiency (n=1), APDS (n=1), and X-linked lymphoproliferative syndrome (n=1). In one patient, a referral for HSCT was recalled based on the outcome of the genetic evaluation. This patient proved to have Ataxia Teleangiectasia, which led to the decision to defer from HSCT. In the patients with ADA2 deficiency and G6PD deficiency, disease specific consultation was initiated.

### Inconclusive Outcomes

#### Risk Factors

Risk factors were identified in 13/165 patients (7.9%) (**Table 1**). The antibody deficiencies category accounted for 7/13 (53.8%) of patients with risk factors. Risk factors were also reported within combined immunodeficiencies (n=2/13; 7.7%), immune dysregulations (n=2/13; 7.7%), and autoinflammatory diseases (n=2/13; 7.7%).

Nine patients were diagnosed with a variant of *TNFRSF13B*. Seven of these patients had a clinical diagnosis of common variable immunodeficiency disorder. *TNFRSF13B* variants in CVID patients were interpreted as contributing to clinical manifestation, and not as genetic diagnosis. Variants in this gene were also identified in one patient in the immune dysregulation group and once in the autoinflammatory disease group. Two patients were diagnosed with a risk factor in *TLR3*. Both patients were categorized in the combined immunodeficiencies group.

#### Carrierships

Carrierships were identified in 25/165 patients (15.2%) and were predominantly found in the categories of antibody deficiencies (n=10/25; 40%), combined immunodeficiencies (n=5/25; 20%) and immune dysregulation (n=5/25; 20%). In three cases, the phenotype of the patient matched the disease for which carriership was detected, raising the suspicion of a second variant on the other allele, which was however not detected by NGS. Specifically, this was the case for a *TCIRG1* variant in a patient with pancytopenia, an *IL36RN* variant in a patient with refractory eczema, and in a *UNC13D* variant in a patient with hemophagocytic lymphiohistiocytosis. No additional evaluations (such as the evaluation for absence of mRNA or a dominant negative role of a heterozygous mutation) were performed in these cases, and a definitive diagnosis could not be made in these patients.

The other carrierships identified in the study cohort were not compatible with the clinical manifestations and were therefore not explicitly mentioned as such.

#### Variants of Unknown Significance

In 38/165 (23%) patients, one or more heterozygous variants of unknown significance in autosomal recessive diseases were reported. In this group, no second pathogenic variants were found and the clinical manifestations were not matching. At the start of the study, there was no consensus on the reporting of VUS within the diagnostic trajectory. Therefore, the number of patients with a reported VUS might be an underrepresentation of the number of patients were a VUS was detected. In four patients, potentially relevant VUS were reported. This was the case for a patient with a homozygous *RAB27A* variant with a clinical diagnosis of FHL. An *NFKB1* variant was found in a patient with recurrent autoimmune hemolytic anemia, antibody deficiency and splenomegaly and a *TRAF3* variant was found in a patient with antibody deficiency.

#### Clinical Implications

In the patients with inconclusive outcomes, SNP arrays were performed in one patient with a risk factor, in three patients with carrierships and in two patients with a VUS. Results revealed a negative outcome. Variants in *NFKB1* (n=1) and *TRAF3* (n=1) were designated as matching with the clinical manifestations. Follow up testing was not performed, and no alterations in clinical management were noted.

### Genotype Without Abnormalities in the IEI Gene Panel

In 64/165 patients (38.8%), no genetic explanation for the clinical manifestations was found during our study (**Table 1**). Most of the patients within this group were clinically diagnosed with antibody deficiencies (n=20/64;31.3%), followed by autoinflammatory diseases (n=14/64;21.9%) and combined immunodeficiencies with associated or syndromic features (n=13/64; 20.3%). Clinical manifestations were diverse in patients without abnormalities in the IEI gene panel, but within the antibody deficiency group, most patients had common variable immune deficiency spectrum disorder.

#### Clinical Implications

In patients without abnormalities identified in the gene panel, the result of the IEI panel did not have specific clinical implications. Clinical management was unchanged and in some cases additional testing was performed, that did not involve further immunological evaluation. Within this group, in two patients, the outcome resulted in the end of the immunological diagnostic trajectory and follow up.

## Discussion

In this study we evaluated a prospective cohort of 165 patients with a suspicion of an inborn error of immunity (IEI) and assessed the outcomes of NGS as an early routine diagnostic tool. Introduction of uniform NGS based IEI diagnostics in The Netherlands resulted in a diagnostic yield of 24.6% in pediatric patients and 9% in adults, resulting in an overall diagnostic yield of 15.2%. This yield varied from 0-42%, depending on immune deficiency disease category, with highest yields found in the disease categories immune dysregulation and phagocyte diseases. In all cases with a conclusive genetic diagnosis, this result had direct clinical implications.

Patients presenting with severe clinical manifestations and life-threatening complications were more likely to receive a conclusive molecular diagnosis, compared to patients with a less severe phenotype. Conclusive genetic diagnoses led to referral for hematopoietic stem cell transplantation (HSCT) in 9 out of 25 (36%) patients, including those with a diagnosis of hemophagocytic lymphohistiocytosis (FHL) and X-linked lymphoproliferative syndrome (XLP2). Especially within these IEIs, adequate and early diagnosis is important, as timely diagnosis and earlier initiation of HSCT is associated with improved outcome (17, 24–30). We did not identify patients with SCID within our study cohort. This might be due to the fact that TREC based screening of SCID was added to the national newborn screening in part of The Netherlands during the study period (31, 32) and by the fact that our study comprised a relatively small sample size. Nevertheless, a significant number of patients was referred for HSCT, thus highlighting the added value of early NGS within routine IEI diagnostics for the more severe IEIs, even after the implementation of newborn screening programs.

We observed that antibody deficiencies represented the majority of our study cohort, with a total of 55/165 (33.3%) patients. This is consistent with previous findings as they comprise the most prevalent symptomatic category of IEI (23, 33) and appear to be more prevalent in Northern European countries than in Asian and African populations (34). In the group of antibody deficiencies, the overall diagnostic yield was 9.1%. This might be attributed to the fact that, for instance, antibody deficiencies such as CVID may be polygenic, with varying penetrance and expressivity, and phenotypic heterogeneity which makes it especially hard to establish a genetic diagnosis (22, 33, 34). This is supported by the fact that carrierships and risk factors were found relatively often in the antibody deficiency group.

In many cases, the outcome of NGS remained inconclusive despite the presence of variants in genes known to be associated with IEIs, as was the case for different VUS, carrierships and risk factors. Guidelines for the interpretation of germline variants are available (21), but interpretation is often complicated by presence of variants with decreased penetrance or milder presentations (22, 34–36). Moreover, rare pathogenic variants in autosomal recessive diseases may be present in population frequency databases in a carrier state (22, 37). Therefore, there is a clear need for additional guidelines enabling more uniform interpretation of inconclusive outcomes, and for additional follow testing of VUS.

In two cases, we found genetic outcomes that did not explain the clinical diagnosis of the patients, as was the case with the *G6PD* variants. The *G6PD* variants reported in our study could have been designated as incidental finding. Nevertheless, *G6PD* has been classified as related to IEI and was therefore included in the IUIS classification (23). Therefore, this variant was not marked as incidental finding, as would be the case for an heterozygous *ATM* variant that might increase the risk of breast cancer (38).

To avoid unnecessary inconclusive outcomes and to help addressing which variants need reporting or require follow up, variant filtering based on clinical phenotype would be of added value. Efforts towards a phenotype based analysis, e.g. using Human Phenotype Ontology (HPO), are currently made as diagnostic yields may increase and become more precise with this technique (39). To make this method available for the IEI setting, initiatives have been developed to curate the HPO for IEI (40). Close collaboration between disease-specific bioinformatics specialists, clinical laboratory geneticists and clinicians, especially in the case of VUS, is also proposed (41).

The diagnostic yields of 24.6% in children and 9% in adults are comparable to the percentages found in other publications, including a study by Arts et al. published in 2019 (15). By contrast, an Iranian study reported a diagnostic yield of 77.8% in 234 CID patients (42), and an Italian study reported a definitive molecular diagnosis in 28.6% of the included patients (11). A systematic review revealed diagnostic yields varying from 15 to 79% (14). Differences in diagnostic yields may be attributed to the severity of symptoms in the patients evaluated and on parental carrier- and consanguinity rates, as the majority of IEI is caused by autosomal recessive conditions (33). The role of country of origin of the patients is also significant, as is supported by the study of Arts et al., reporting a genetic diagnosis in 14% and 57% of patients from Europe and Saudi Arabia, respectively (15). The specific IEI subcategory of the patients being studied may also influence diagnostic yield, as shown by studies by Abolhassani et al. (2018) and Cifaldi et al. (2019) including higher percentages of CID and SCID patients, which may have increased the diagnostic yield (11, 42). Finally, age of the patients under investigation plays a role, as shown by the study of Cifaldi et al. (2019) who included more infants compared to our study, and who reported a diagnostic yield of 28.7%.

All Dutch genome diagnostic centers have implemented NGS-based diagnostic tools (43). Different studies emphasized the challenges that come with NGS based diagnostics, such as complex informed consent procedures, and the interpretation and management of variants of which the clinical significance is unknown (44, 45). Vrijenhoek et al. showed that assessing the effects of NGS based diagnostics is not solely a matter of overcoming the abovementioned challenges (45). To provide high-quality genomic care, it is important to make every step within the process a high-functioning, nationwide interplay between specialists from all disciplines involved. This study offered the unique opportunity to coordinate and manage the interplay between multidisciplinary expert teams and the genetic evaluation of IEI patients at the same time, and to assess the outcomes of both in the form of different patient outcomes of all genome diagnostic centers in the Netherlands.

We took different steps to make the process of NGS based IEI diagnostics uniform and feasible across all genome diagnostic centers. First, a uniform NGS based IEI panel was used to evaluate all patients. Prior to the implementation of this technique as part of the standard evaluation in patients with a clear suspicion of IEI, different NGS based IEI panels were offered in the participating university hospitals. At introduction of the technique in the standard work up, a uniform IEI panel was agreed upon, based on the IUIS classification at that moment. In three-monthly consensus meetings, this panel was expanded for all participation genome centers from 360 genes to 426 genes at the end of the study. We further performed a national external quality assessment (19) to ensure a uniform diagnostic pipeline and variant interpretation throughout the study, thereby decreasing the risk of technical biases in our study. Finally, the study was based on results generated by collaboration between clinicians and clinical laboratory geneticists, and therefore mimics the setting of daily clinical practice and thus represents the routine diagnostic trajectory. It also highlights the importance of the intensive interaction between multidisciplinary expert teams of clinical laboratory geneticists, clinical geneticists, medical specialists, bioinformaticians, and data analists.

We analyzed the results of an NGS based IEI panel, applied to WES or WGS based sequencing data. The advantage of WES and WGS data is the opportunity to further evaluate those patients in whom no conclusive diagnosis was made in the IEI panel, by further analyzing the data already generated (46), as was done in two cases and resulted in the detection of Gaucher disease in one patient. Another advantage of using a WES or WGS based approach is that during clinical follow up, analysis of novel IEI related genes is relatively easy, as will also be performed in this cohort in the near future. WES and WGS data also enable the detection of CNVs. CNV analysis was routinely performed in one diagnostic center in this study, evaluating a total of 49 patients, yielding the detection a duplication of the MEFV gene without known clinical significance. Although NGS can be used to detect somatic variants, the depth of sequencing we applied with WGS (~30x) and WES (~75x) did not allow to detect somatic variants with a low allele frequency. We found one *GATA2* variant that was considered to be somatic, as we did not detect it in other tissues tested in this patient. Theoretically, a WGS based approach could yield higher percentages of conclusive diagnoses compared to WES based approach, as it enables the detection of non-coding variants (46). To determine the clinical relevance of non-coding variants, further functional testing is often needed, however. The disadvantages of WES and WGS analyses as a first diagnostic step include the fact that these analyses are time consuming and may yield significant numbers of inconclusive findings and the possibility of detecting unsolicited findings (44).

We found that the time to diagnosis, defined as time between first medical consultation for a IEI and diagnosis, was shorter in children than in adults. This may be explained by the fact that some children had severe disease features at presentation leading to an immediate suspicion of severe IEI, with subsequent rapid diagnostic work up using trio WES, in some cases leading to a turn-around time for genetics of six days. It may also be explained by the fact that in the adult population, several patients were included who had a long and ongoing evaluation of IEI, for whom the NGS based panel finally provided a diagnosis. This underlines the importance of this type of diagnostics even for adult patients, as a definitive diagnosis often ends a diagnostic odyssey, enables specific therapy and family screening. For samples evaluated in the routine setting, no statistically significant differences between WES and WGS in TAT or overall time to diagnosis were found.

A limitation of our study is that the results were mainly based on the application of a specific gene panel, that was updated throughout the study period. Therefore we may have missed variants in specific genes in patients who were included within the earlier stages of the study. Also, further evaluation of inconclusive outcomes to assess biological relevance of a VUS were not a routine procedure, and this may have led to an underestimation of the number of conclusive diagnoses

In conclusion, the early application of an NGS based IEI panel proved to be a highly valuable routine diagnostic tool. We found highest yields in patients under 18 years and in patients with more severe disease phenotypes. More focus should be on providing guidelines and criteria to increase the numbers of conclusive outcomes, as well as guidelines on how to manage VUS. Moreover, with the rapidly evolving field of IEI related genes, assessing genetic defects within these patients should be an ongoing process; periodic reanalysis of the WES data is advisable. Finally, to provide more insight into the effectiveness of NGS as a whole, future studies should be focused on the health economic effects of early NGS as guidelines as policy decisions cannot solely be based on clinical outcomes of the diagnostic tool.

## List of Genes

*ADA*, Adenosine deaminase; *ADAR*, Adenosine deaminase, RNA-specific; *ATM*, ATM serine/threonine kinase; *BTK*, Bruton tyrosine kinase; *CD2BP1*, CD2 binding protein 1; *CFTR*, Cystic fibrosis transmembrane conductance regulator; *DOCK8*, Dedicator of cytokinesis 8; *FAS*, Fas cell surface death receptor; *GINS1*, GINS complex subunit 1; *IFIH1*, Interferon-induced helicase C domain-containing protein 1; *IKBKB*, Inhibitor of nuclear factor kappa-b kinase, subunit beta; *IL6ST*, Interleukin 6 Cytokine Family Signal Transducer; *LTBP3*, Latent transforming growth factor beta binding protein 3; *MBL*, Mannose-binding lectin; *MEFV*, Familial Mediterranean Fever gene; *MKL1*, Megakaryoblastic leukemia 1; *MPO*, Myeloperoxidase; *NFKB1*, Nuclear factor kappa-b, subunit 1; *NFKB2*, Nuclear factor kappa-b, subunit 2; *NLRP12*, NLR family, pyrin domain-containing 12; *PIK3CD*, Phosphatidylinositol 3-kinase, catalytic, delta; *PLEKHM1*, Pleckstrin homology domain-containing protein, family M, member 1; *PRF1*, Perforin 1; *PSTPIP1*, Proline/serine/threonine phosphatase-interacting protein 1; *RFXANK*, Regulatory factor x, Ankyrin repeat-containing; *SBDS*, Shwachman-Bodian-Diamond Syndrome protein; *STAT3*, Signal transducer and activator of transcription 3; *STX11*, Syntaxin11; *TCF3*, Transcription factor 3; *TNFRSF13B*, Tumor necrosis factor receptor superfamily, member 13B; *TRNT1*, tRNA-nucleotidyltransferase 1; *UNC13D*, Unc-13 homolog D; *XIAP*, Inhibitor of apoptosis, X-linked; *TRAF3*, TNF receptor-associated factor 3.

## Data Availability Statement

The original contributions presented in the study are included in the article/**Supplementary Material**. Further inquiries can be directed to the corresponding author.

## Ethics Statement

The studies involving human participants were reviewed and approved by Medical Ethical Board of the Erasmus MC, Erasmus University Rotterdam, Rotterdam, The Netherlands. Written informed consent to participate in this study was provided by the participants’ legal guardian/next of kin.

## Genetics First for Primary Immunodeficiency Disorders Consortium

**Aerde van, KJ.** Department of Pediatric Infectious Diseases and Immunology, Amalia’s Children Hospital, Radboud University Nijmegen Medical Centre, Nijmegen, The Netherlands

**Altenburg, J.** Department of Respiratory Medicine, Amsterdam University Medical Center, Amsterdam, the Netherlands.

**Armbrust W.** Department of Pediatric Rheumatology and Immunology, Beatrix Children’s Hospital, University Medical Center Groningen, Groningen, The Netherlands.

**Barendregt, BH.** Department of Immunology, Erasmus MC, University Medical Center Rotterdam, Rotterdam, Netherlands.

**Berg van den, JM**. Department of Pediatric Hematology, Immunology and Infectious Diseases, Emma Children’s Hospital, Amsterdam University Medical Center, Amsterdam, The Netherlands

**Bredius, RGM**. Department of Pediatrics and Laboratory for Pediatric Immunology, Willem-Alexander Children’s Hospital, Leiden University Medical Center, Leiden, Netherlands

**Buddingh, EP.** Willem-Alexander Children’s Hospital, Department of pediatrics, Leiden University Medical Center, the Netherlands

**Burg van der, M.** Department of Pediatric and Laboratory for Pediatric Immunology, Leiden University Medical Center, Leiden, The Netherlands

**Ellerbroek, PM.** Department of Infectious Diseases, University Medical Center Utrecht, Utrecht, The Netherlands

**Ernst, RF**. Department of Genetics, Division Laboratories, Pharmacy and Biomedical Genetics, University Medical Center Utrecht, Utrecht, The Netherlands

**Fraaij, PLA**. Department of Pediatric Infectious Diseases, Immunology and Rheumatology, Sophia Children’s Hospital, Erasmus Medical Center, Rotterdam, The Netherlands

**Hermans, M.** Department of Internal Medicine, Division of Clinical Immunology, Department of Immunology, Erasmus MC, Rotterdam, The Netherlands.

**Hoischen, A**. Department of Human Genetics, Nijmegen Center for Molecular Life Sciences, Radboud University Medical Centre, Nijmegen, The Netherlands; Radboud Expertise Center for Immunodeficiency and Autoinflammation; Department of Internal Medicine, Radboud University Medical Center, Nijmegen, The Netherlands.

**Hout van der, AH.** Department of Genetics, University Medical Center Groningen, University of Groningen, Groningen, The Netherlands

**Jansen, MHA.** Department of Pediatric Immunology and Infectious Diseases, Wilhelmina’s Children Hospital, University Medical Center Utrecht, Utrecht, The Netherlands

**Jolink, H.** Department of Hematology, Leiden University Medical Center Department of Infectious Diseases, Leiden University Medical Center

**Jonkers, RE.** Department of Respiratory Medicine, Amsterdam University Medical Center, Amsterdam, the Netherlands.

**Laar van, JAM.** Department of Internal Medicine, Division of Clinical Immunology, Department of Immunology, Erasmus MC, Rotterdam, The Netherlands

**Leeuw de, K.** Department of Rheumatology and Clinical Immunology, University Medical Center Groningen, Groningen, the Netherlands

**Legger, GE.** Department of Pediatric Rheumatology and Immunology, Beatrix Children’s Hospital, University Medical Center Groningen, Groningen, The Netherlands

**Leijten, EFA**. Department of Rheumatology & Clinical Immunology, University Medical Center Utrecht, Utrecht, The Netherlands

**Limper, M.** Department of Rheumatology and Clinical Immunology, University Medical Center Utrecht, Utrecht, the Netherlands

**Lindemans, CA.** Department of Pediatrics, Wilhelmina Children’s Hospital, University Medical Center Utrecht, Utrecht, The Netherlands; Princess Maxima Center, Utrecht, The Netherlands.

**Oever ten, J**. Department of Internal Medicine, Radboud University Medical Center, Radboud Center for Infectious Diseases, Nijmegen, The Netherlands

**Pieterse, M**. Department of Human Genetics, Nijmegen Center for Molecular Life Sciences, Radboud University Medical Centre, Nijmegen, The Netherlands

**Rombach, SM.** Department of Internal Medicine, Allergy and Clinical Immunology, Erasmus MC University Medical Center, Rotterdam, the Netherlands

**Rossum van, AMC**. Department of Pediatric Infectious Diseases, Immunology and Rheumatology, Sophia Children’s Hospital, Erasmus Medical Center, Rotterdam, The Netherlands

**Rutgers, A**. Department of Rheumatology and Clinical Immunology, University Medical Center Groningen, The Netherlands

**Santen, GWE.** Department of Clinical Genetics, Leiden University Medical Center (LUMC), Leiden, Netherlands

**Schölvinck, EH** Department of Pediatric Infectious Diseases and Immunology, Beatrix Children’s Hospital, University Medical Center Groningen, Groningen, The Netherlands

**Simon, A.** Department of General Internal Medicine, Radboud University Medical Center, Nijmegen, The Netherlands

**Stol, K**. Department of Pediatric Infectious Diseases and Immunology, Amalia’s Children Hospital, Radboud University Nijmegen Medical Centre, Nijmegen, The Netherlands

**Vervenne RML**. Department of Genome Diagnostics, Amsterdam University Medical Center, Amsterdam, The Netherlands

## Author Contributions

We state that the co-authors have performed the following tasks for the paper: KE: Data collection, overall writing the paper. MH: Generation of data, making it ready to analyse, education of the PhD student on interpretation variants, and co-writing. IH: Generation of data, making it ready to analyse and co-writing. AS: Generation of data, making it ready to analyse and co-writing. EZ-H: Generation of data, making it ready to analyse and co-writing. LV: Generation of data, making it ready to analyse and co-writing. HL: Inclusion/selection patients, entering data, co-writing. SH: Inclusion/selection of patients, entering data, co-writing. MD: Inclusion/selection of patients, entering data, co-writing. FV: Inclusion/selection of patients, entering data, co-writing. JP: Inclusion/selection of patients, entering data, co-writing. DB: Inclusion/selection of patients, entering data, co-writing. VD: Inclusion/selection of patients, entering data, co-writing. CV: Inclusion/selection patients, entering data, co-writing. AV: Inclusion/selection patients, entering data, co-writing. AL: Responsible for the process of handling incoming samples, co-writing. KA: Generation of gene panel, co-writing. PH: Inclusion/selection patients, co-writing. GB: Inclusion/selection patients, co-writing. TK: Inclusion/selection patients, co-writing. GF: Co-writing. MG: Supervision, education of KE on sequence variants, overall guidance and writing. JM: Supervision, overall guidance and writing. All authors contributed to the article and approved the submitted version.

## Funding

This study is part of the Genetics First: next-generation sequencing for cost-effective PID diagnostics project. This project is funded by ZonMw (grant number 846002001).

## Conflict of Interest

The authors declare that the research was conducted in the absence of any commercial or financial relationships that could be construed as a potential conflict of interest.

## Publisher’s Note

All claims expressed in this article are solely those of the authors and do not necessarily represent those of their affiliated organizations, or those of the publisher, the editors and the reviewers. Any product that may be evaluated in this article, or claim that may be made by its manufacturer, is not guaranteed or endorsed by the publisher.

## Abbreviations

ACMG, American College of Medical Genetics; AD, Autosomal dominant; AID, Activation induced cytidine deaminase; ALPS, Autoimmune lymphoproliferative syndrome;APDS, Activated PI3K delta syndrome; AR, Autosomal recessive;AT, Ataxia telangiectasia; CBC, Complete blood count; CGD, Chronic granulomatous disease; CID, Combined immunodeficiency; CVID, Common variable immunodeficiency disorder; DADA2, Deficiency of Adenosine Deaminase 2; EDA-ID, Anhidrotic ectodermodysplasia with immunodeficiency; FHL2/3/4, Familial hemophagocytic lymphohistiocytosis type 2/3/4; GoF, Gain-of-Function; HLH, Hemophagocytic lymphohistiocytosis; HSCT, Haematopoietic stem cell transplantation; ID, Intellectual Disability; IEI, Inborn errors of immunity; IGRT, Immunoglobulin replacement therapy; IUIS, International Union of Immunological Societies; NGS, Next-Generation Sequencing; PAPAS, Pyogenic Arthritis, Pyoderma gangrenosum and Acne syndrome; PID, Primary Immunodeficiency; SCID, Severe combined immunodeficiency; TAT, Turn-around time; VCF, Variant call format; WES, Whole-Exome Sequencing; WGS, Whole-Genome Sequencing; XLA, X-linked agammaglobulinemia; XLP, X-linked lymphoproliferative syndrome.
