# Supplementary material for: Implementation of Early Next-Generation Sequencing for Inborn Errors of Immunity: A Prospective Observational Cohort Study of Diagnostic Yield and Clinical Implications in Dutch Genome Diagnostic Centers
Source: Front Immunol. 2021 Dec 21;12:780134. doi: 10.3389/fimmu.2021.780134 (PMC8724043; doi:10.3389/fimmu.2021.780134)
Supplement: Supplementary file 2 [file Table_2.docx]

**Table S2.** Basic characteristics of study cohort

|  | **Total (N=165)** |
| --- | --- |
| Median age at first consultation for of IEI (IQR), years | 21 (38) |
| Male sex, n (%) | 74 (44,8) |
| **NGS-method, n (%)** | |
| Whole-genome sequencing | 65 (39,4) |
| Whole-exome sequencing | 100 (60,6) |
| **Clinical diagnosis at inclusion based on IUIS classification, n (%)** |  |
| Combined B- and T-cell deficiencies | 11 (6,7) |
| Combined immunodeficiencies with associated or syndromic features | 25 (15,2) |
| Predominantly antibody deficiencies | 47 (28,5) |
| Diseases of immune dysregulation | 35 (21,2) |
| Phagocyte diseases | 10 (6,1) |
| Defects in intrinsic and innate immunity | 8 (4,8) |
| Autoinflammatory diseases | 14 (8,5) |
| Complement deficiencies | 2 (1,2) |
| Bone marrow failure | 0 (0,0) |
| Positive family history with clinical laboratory abnormalities | 11 (6,7) |
| Other | 2 (1,2) |
| Time between first visit and diagnosis (median, IQR), days | 121 (282) |
